# Agent-based models of human response to natural hazards: systematic review of tsunami evacuation

**DOI:** 10.1007/s11069-022-05643-x

**Published:** 2022-10-05

**Authors:** Karel Mls, Milan Kořínek, Kamila Štekerová, Petr Tučník, Vladimír Bureš, Pavel Čech, Martina Husáková, Peter Mikulecký, Tomáš Nacházel, Daniela Ponce, Marek Zanker, František Babič, Ioanna Triantafyllou

**Affiliations:** 1 Faculty of Informatics and Management, University of Hradec Králové, 500 03 Hradec Králové, Czech Republic; 2 Faculty of Electrical Engineering and Informatics, Technical University of Košice, 042 00 Košice, Slovakia; 3 Department of Geology and Geoenvironment, National & Kapodistrian University of Athens, 15772 Athens, Greece

**Keywords:** Agent-based model, Natural hazard, Tsunami, Simulation, Crowd motion, Evacuation

## Abstract

This systematic review provides a comprehensive overview of tsunami evacuation models. The review covers scientific studies from the last decade (2012–2021) and is explicitly focused on models using an agent-based approach. The PRISMA methodology was used to analyze 171 selected papers, resulting in over 53 studies included in the detailed full-text analysis. This review is divided into two main parts: (1) a descriptive analysis of the presented models (focused on the modeling tools, validation, and software platform used, etc.), and (2) model analysis (e.g., model purpose, types of agents, input and output data, and modeled area). Special attention was given to the features of these models specifically associated with an agent-based approach. The results lead to the conclusion that the research domain of agent-based tsunami evacuation models is quite narrow and specialized, with a high degree of variability in the model attributes and properties. At the same time, the application of agent-specific methodologies, protocols, organizational paradigms, or standards is sparse.

**Supplementary Information:**

The online version contains supplementary material available at 10.1007/s11069-022-05643-x.

## Introduction

Natural disasters, in general, have the potential to impact inhabited areas heavily and can potentially cause many fatal injuries and massive damage to infrastructure and property. Our research is focused primarily on the consequences of tsunami waves, which usually follow severe earthquakes and affect large coastal areas. Given the nature of tsunamis, there is usually an extremely limited time window to handle the evacuation of people to shelters located either at higher altitudes or further from the zones of wave impact. During this limited time, it is necessary to maximize the efficiency of evacuation procedures and minimize the risks of endangering human lives. This is one of the main features of tsunami evacuation models that distinguishes our research from similar but more general studies of evacuation models (Kaur and Kaur 2022).

In order to find the best and most efficient practices, it is necessary to study evacuation processes in detail using various disaster scenarios under different settings. Mathematical models represent one of the standard approaches to examining patterns of group behavior. On the other hand, approaches based on real observations and experiments, e.g., during evacuation drills (Chen et al. 2022) provide valuable data that can be used to tune the parameters of computer models. These models, in turn, enable massive in silico testing of alternative scenarios, including extremely rare or risky ones.

This systematic review presents a detailed analysis of agent-based models (ABMs) used to study evacuation processes under various circumstances. The review focuses on the last decade of research in this domain (2012–2021) to take into account current results in particular. This choice is also supported by the fact that primary sources record the first works on the subject under review also in this time period (Web of Science from 2012, Scopus from 2010). Secondary sources, such as Google Scholar, record the first relevant article in 2001, but the majority of publications (about 90%) again fall into the last decade. We analyzed 171 papers or articles obtained from primary sources and belonging to the selected period, and after applying the discrimination criteria using the PRISMA methodology (Page et al. 2021), 53 of them were selected for detailed full-text analysis.

The focus of this review, specifically on ABM applications, is motivated by the scalability, adaptability, and customizability of the agent-oriented approach which has been proven in various domains (Bureš and Tučník 2014). Agents are capable of autonomous decision-making and social interactions, and can conclusively simulate human behavior during disasters, including phenomena such as panic, errors in judgment, health conditions, and weather. Since ABMs are usually constructed using a bottom-up approach, the overall performance can be studied under randomized/customized conditions at the global level (Bonabeau 2002). As such, agent-based (social) simulations have the potential to contribute meaningfully to a better understanding of the large-scale processes related to evacuation procedures and the mitigation of risks related to disasters. This review is also related to our previous research (Nacházel et al. 2021) focused on analyzing tsunami-related data and datasets. At the same time, the possibilities of ontologies as perspective methods of representation and formal description of the observed domain are investigated (Babič et al. 2022).

This systematic review is divided into six sections. The second section describes the methodology of the research and research questions that we aim to answer in this review. The third part focuses on the technical properties of ABMs (with special attention given to features specific to an agent-oriented approach), such as agent-based modeling tools, the validity of the models, and other technical parameters. The fourth section describes the model attributes, for example, the main purpose of the models, their special features, a representation of the environment, input and output data, and other model-related details. The fifth part is dedicated to the discussion and summarization of the results. Finally, the sixth section concludes this review.

## Methodology

The present study is focused on an agent-based simulation of a tsunami evacuation. A review based on PRISMA methodology (Page et al. 2021) was conducted. We aim to answer the following four research questions:

1. What were agent-based models developed in relation to a tsunami evacuation?
2. How were such ABMs specifically designed?
3. Which goals or performance criteria were ABMs trying to pursue?
4. What are the research opportunities and gaps in the area of agent-based evacuation models of tsunami scenarios?

An initial cross-search was conducted using scientific databases Web of Science and Scopus in July–August 2021. The selection criteria and data collection strategy focused on agent-based simulation, tsunami, and evacuation. The review included full texts published in English between 2012 and 2021. Initially, standard search tools within WoS and Scopus were used, which returned all articles meeting the specified criteria. By applying the search to all fields of the databases, however, results were also found that mentioned the defined characteristics only marginally or partially—for example, in references to sources in the introductory or research section, or as “future work.”

The abstracts, keywords, and practical chapters of these articles were subsequently screened to pre-exclude articles we identified as not meeting the search criteria (Table 1).

**Table 1:** Inclusion criteria

Criterion	Requirement
Language	English
Type of paper	Journal article, conference paper, preprint
Publication	01/2012–07/2021
Topic	Agent-based social simulation for investigation of tsunami evacuation process

**Fig. 1:**
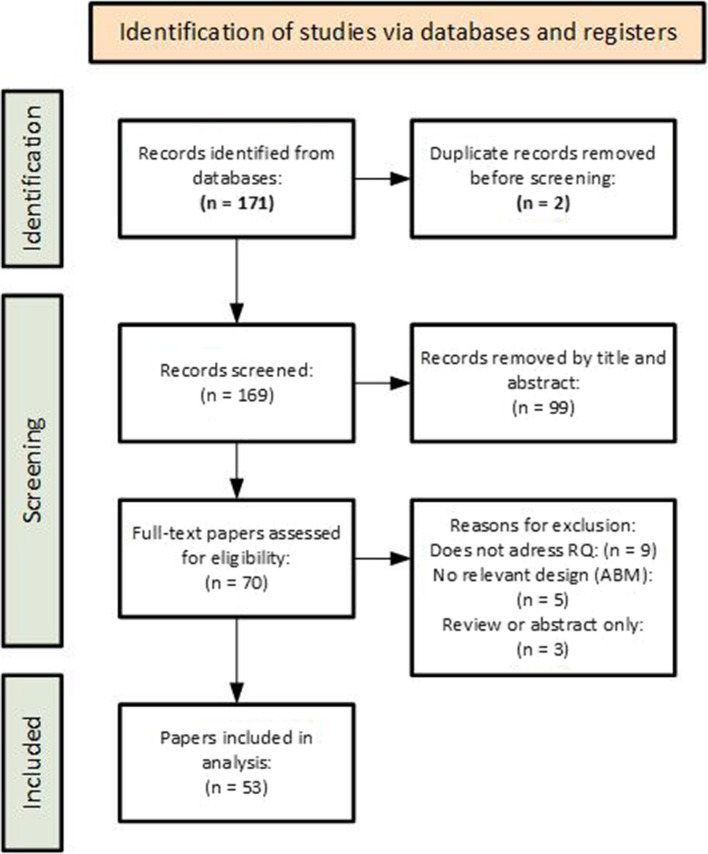
PRISMA flow diagram

Thus, 171 articles were initially identified from primary scientific databases. After removing duplicates and irrelevant papers based on orientation screening (by title, abstract, and non-research chapters), 70 papers were advanced to the full-text evaluation phase for eligibility. The result of this preparatory phase was 53 papers suitable for both qualitative and quantitative analysis. The entire process is illustrated in Fig. 1 and a complete list of selected papers is given in Online Resource 1.

Two lists of paper characteristics are defined for the implementation of qualitative and quantitative analysis. List A focuses on the technical properties of papers (agent-based modeling-related issues and standards), resulting in descriptive statistics (see Sect. 3):

1. What agent-based modeling tool or programming language was used?
2. Are the source code and documentation available?
3. Are any scenarios defined?
4. Are experiments presented?
5. Were statistical methods applied?
6. Was a sensitivity analysis conducted?
7. What validation method was used?

List B focuses on a more detailed analysis of the models used in the articles (see Sect. 4):

1. What geographical area is simulated by a model?
2. What is the general purpose of the model?
3. Are any special features/aspects/issues of evacuation explored?
4. How is the environment represented?
5. What types of agents are defined, what are their attributes?
6. What are the agents’ attributes, if specified?
7. What input data/variables are used in the models?
8. What output data/variables/measures does the model produce?
9. Are there any simplifications or features omitted from the model and/or proposed by the authors for future work?
10. Are there any specific algorithms used by agents?

Each paper was reviewed by at least two reviewers.

## Technical properties of ABM models

The descriptive analysis focused on the technical properties of the selected texts (as listed in Sect. 2, List A), and, in addition to the binary outputs (condition met/not met), the occurrences of specific responses were also recorded (e.g., types of ABM tools and scenarios/experiments used in simulations).

### Item A1: What agent-based modeling tool or programming language was used?

With this question, we focused on determining which software tools were used in selected articles to implement the agent models. Dozens of different tools have been created and used to varying degrees over the years (Nikolai and Madey 2009). Currently, the most well-known tools in application domains such as traffic simulations, GIS, mobility planning, and evacuation are summarized in Table 2.

**Table 2:** Software tools in selected ABMs application domains (Abar et al. 2017)

ABMs application domain	ABMs software tools
Social & natural sciences, dynamic computational systems, business, marketing, economics, ecology, healthcare, planning & scheduling, enterprise and organizational behavior, **traffic situations** (avoidance of traffic jams, light control, route choice)	Agent Factory, AgentScript, AgentSheets, **AnyLogic** (2D/3D), AOR Simulation, Ascape, BehaviourComposer (2D/3D), Brahms, Cormas, CybelePro, Echo, Envision, Eve, ExtendSim (2D/3D), FLAME, FlexSim, Framsticks (2D/3D), GALATEA, **GAMA** (2D/3D), GROWLab, HLA_Agent, HLA_RePast, IDEA, Insight Maker, JAMSIM, Janus, JAS, JAS-mine, jES, LSD (2D/3D), **MASON** (2D/3D), MASS, Mathematica® (Wolfram), **MATSim**, Mimosa, MIMOSE, MOBIDYC, Modgen, **NetLogo** (2D/3D), Pandora, PDES-MAS, PS-I, Repast-J/Repast-3, Repast HPC, **Repast Simphony** (2D/3D), SeSAm, SimAgent, SimBioSys, SimEvents (MATLAB®), Simio (2D/3D), SimJr, SimSketch, Simul8, SOARS, StarLogo, Sugarscape, Swarm, UrbanSim, VisualBots, VSEit
**Geographic information system** (GIS), geographic automata system (GAS)	Cormas, Envision, **GAMA** (2D/3D), Insight Maker, **MATSim**, OBEUS, Pandora, Repast-J/Repast-3, Repast HPC, **Repast Simphony** (2D/3D), SOARS, TerraME
Aviation, flight, or air-traffic control, **ground transportation/mobility planning systems**	CybelePro, D-OMAR, ExtendSim (2D/3D), FlexSim (2D/3D), MACSimJX, **MATSim**, Mobility testbed, SimEvents (MATLAB®), Simio (2D/3D), SimJr, Swarm, UrbanSim
**Urban planning** (accessibility studies with dynamic populations)	**AnyLogic** (2D/3D), CRAFTY, Envision, **GAMA** (2D/3D), JAS-mine, Modgen, OBEUS, UrbanSim
Microscopic pedestrian crowd or mapping passenger flow (market improvement & **evacuation of buildings**)	Brahms, HLA_Agent, HLA_RePast, PDES-MAS, PedSim, Repast-J/Repast-3, Repast HPC, **Repast Simphony** (2D/3D), SeSAm, Simio (2D/3D)

**Fig. 2:**
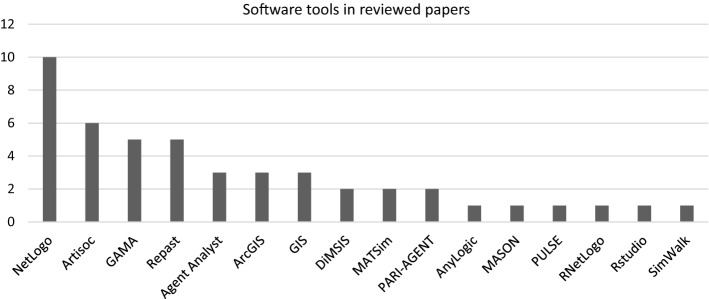
Frequency of occurrence of individual software tools in the analyzed texts

In 16 out of 53 analyzed papers (30%), the authors did not mention any specific tool or platform used in their research. In other texts, the use of the universal academic NetLogo platform (10x) prevailed over specialized tools Repast Simphony (5x), GAMA (5x), and MATSim (2x) (see Fig. 2).

### Item A2: Are the source code and documentation available?

Only 5 of the 53 papers were accompanied by source code (Aguilar et al. 2017; Nakanishi et al. 2020; Naqvi 2017; Slucki and Nielek 2015; Wijerathne et al. 2013) and only one pseudocode (Poulos et al. 2018) was found. This means that approximately 90% of the models are virtually impossible to replicate and the published results cannot therefore be at least quantitatively verified. This finding is in accord with the results of previous studies (Schulze et al. 2017).

### Item A3: Are any scenarios defined?

In 36 of the papers, the scenarios are defined, and in 17, they are not. Because the multi-agent system can be used to model human behavior and various “what if” scenarios, it can also be used to model and simulate multiple situations that are difficult to test in real life for security reasons. The results then illustrate the way people behave in the proposed situation and can provide a reliable and credible conclusion corresponding to real-time scenarios. Scenarios can be based on various complex patterns of agent behavior—from simple reactive interaction with the environment to complicated and realistic behavior controlled by artificial intelligence (Sharma et al. 2018) (Table 3).

**Table 3:** Types of scenarios

Scenario	Paper
Evacuation with and without collisions	Alam and Habib (2020)
Different number of COVID-19 positive cases	Callejas et al. (2020)
Distribution of debris in the city depending on the magnitude of the earthquake	Castro et al. (2018)
Morning congestion influenced by damage caused by an earthquake	Castro et al. (2019)
Late-night time scenario, reaction delay, fatigue time	Faucher et al. (2019, 2020)
Horizontal and vertical evacuation	Hatayama et al. (2019)
The effectiveness of the capacity and position of the evacuation buildings	Ito et al. (2020)
Tsunami up to 4 m high with propagation time from the source region to the Vietnamese coast estimated at about 2 h	Le et al. (2013, 2014, 2015, 2017)
A worst-case scenario where all the daytime evacuees are in the public space	León et al. (2020)
Comparison of the current urban tsunami emergency scenario with the proposed situation during day and night scenarios	León and March (2014a, b, 2016)
Worst seismic scenario for Central Chile, according to the 1730 event (Mw 9.1–9.3 earthquake)	León et al. (2021)
Worst seismic scenario for Central Chile, according to the 1730 event + daytime/nighttime scenario	León et al. (2018)
Multiple scenarios (nighttime, late evacuation, vehicle–pedestrian interactions)	Mas et al. (2015)
Multiple scenarios (the failure of evacuation links, cars/pedestrians ratio, worst-case scenario)	Mostafizi et al. (2017)
Multiple scenarios (milling time, horizontal/vertical evacuation, shelter location)	Mostafizi et al. (2019a, b)
Multimodal evacuation (walking/driving, milling time, walking/driving speed)	Mostafizi et al. (2019a, b)
Knowledgeable pedestrians and followers response to a tsunami alert	Nguyen et al. (2012)
Spontaneous/signposted/enhanced urban-environment evacuation	Sahal et al. (2013)
Individual and organized groups evacuation	Slucki and Nielek (2015)
Day and night evacuation with horizontal or horizontal/vertical policy	Solís and Gazmuri (2017)
Population scenario combined with the height of the road embankment	Takabatake et al. (2020a, b, c)
Evacuation destination and car usage rate combinations	Takabatake et al. (2020a, b, c)
Multiple scenarios (daytime/night time, summer/winter, earthquake magnitude)	Takabatake et al. (2020a, b, c)
Seven different earthquake scenarios and six different evacuation scenarios	Takabatake et al. (2018)
Seventeen scenarios based on moving speed, visitors behavior, and number of visitors	Takabatake et al. (2017)
Four scenarios with different number of evacuation shelters and their capacity	Usman et al. (2017)
Three scenarios based on agents’ knowledge of evacuation plan and the means they move	Wafda et al. (2013)
Multimodal evacuation simulation for a near-field tsunami scenario	Wang et al. (2016)
Tsunami evacuation and risk assessment in addition with different seismic damage to the transportation network, pedestrian–vehicle interaction, and pedestrian speed adjustment	Wang and Jia (2021)
32 transport demand scenarios (residential/employee, daytime/nighttime)	Wood et al. (2020)
No tsunami scenario	Aguilar et al. (2017) Helton et al. (2013) Ishida et al. (2013), Ishida and Hashimoto (2015) Jumadi and Quincey (2016) Karbovskii et al. (2014, 2015) Katayama et al. (2019) Kunwar et al. (2014) Makinoshima et al. (2018) Medina et al. (2016) Nakanishi et al. (2020) Naqvi (2017) Poulos et al. (2018) Tagg et al. (2016) Wijerathne et al. (2013, 2018)

### Item A4: Are experiments presented?

If we consider the framework scenarios, the next logical step in modeling is to focus on specific experiments. From the analyzed articles, it is clear that both scenarios and experiments are given paramount attention by the authors.

In 42 of the selected papers, experiments are presented, and only in 7 they are not; in addition, in only 2 of the papers are the final results given (Aguilar et al. 2017; Alam and Habib 2020), and the final 2 describe the results briefly or in general (Mas et al. 2015; Slucki and Nielek 2015) (Table 4).

**Table 4:** Types of experiments

Experiment	Paper
Different configurations of shelters	Nakanishi et al. (2020)
Evacuation by foot or by car, walking speed	Mostafizi et al. (2019a, b)
Different speed of pedestrians	Faucher et al. (2020)
Early or delayed evacuation	Takabatake et al. (2020a, b, c)
Spontaneous, signposted, and enhanced urban-environment evacuation	Sahal et al. (2013)

### Item A5: Were statistical methods applied?

In 19 papers from the analyzed set, statistical methods were applied, while in 34 they were not; in addition, most authors provided simple descriptive statistics (bar graphs), or they state that due to the fact that multi-agent models are stochastic in nature, it is necessary to repeat the simulations many times and use only the mean values of the results.

### Item A6: Was a sensitivity analysis conducted?

Among the papers studied, 39 did not specify any method of sensitivity analysis, whereas in 11 publications (Alam and Habib 2020, Kunwar et al. 2014, Le et al. 2013, 2014, 2015, 2017, Mostafizi et al. 2017, 2019a, b, Poulos et al. 2018; Solís and Gazmuri 2017) some mentions of the analysis are given. In one paper, the authors present the analysis as “future work” (Makinoshima et al. 2018), and in (Takabatake et al. 2020a, b, c) the “effects of change of behavior on mortality rate” are discussed, whereas in (Wang et al. 2016) “model sensitivity to critical depth and model sensitivity to s and *r*” is presented.

### Item A7: What validation method was used?

In 39 of the papers, no explicit validation method was specified. A comparison with real-world data is presented in (Castro et al. 2019; Katayama et al. 2019; Sahal et al. 2013; Takabatake 2020a, b), whereas comparison with other models is less common (Alam and Habib 2020; Faucher et al. 2020). The use of mobile phone data to calibrate the ABM outcomes (León et al. 2021) and video analysis (Poulos et al. 2018) or comparison with a shelter plan analysis (Usman et al. 2017) are other validation methods applied.

## Analysis of model attributes

This section of the review focuses on a detailed analysis of the model attributes, listed specifically in Sect. 2, List B.

### Item B1: What geographical area is simulated by a model?

The majority of the models described in papers can be perceived to a certain extent as case studies for specific regions. This is a reasonable approach because it indirectly validates the model results to a certain extent and improves the applicability of the new knowledge obtained from the simulation results. The research is usually focused on areas where tsunamis are a frequent phenomenon, such as coastal regions of Southeast Asia, the western coast of South America or Middle America, and the Mediterranean Sea. This can potentially be important for other parameters of the model settings; therefore, this parameter was included in this study (Table 5).

**Table 5:** Number of studies by geographic locations

State	Region/City/Location	Number	State	Region/City/Location	Number
Japan	Kochi	2	Chile	Iquique	7
	Osaka	2		Vina del Mar	2
	Tanabe city	1		Coquimbo-La Serena	1
	Tanabe/Kamiyashiki	1		Talcahuano	2
	Tanabe/Katamachi	1		Valparaiso	1
	Takamatsu	1	UK	Lincolnshire coast	1
	Kesennuma	1		Norfolk coast	1
	Tokyo	1		Humberside	1
	Kamakura	3		Bath	1
	Shinguu	1		Somerset coast	1
	Sendai/Arahama	1		Chichester	1
	Natori	1		Wakefield	1
	Kamaishi	1		Stirling Council	1
France	Mediterranean coast	1		Milton Keynes	1
Russia	St. Petersburg	2		Bradford	1
New Zealand	Christchurch	1		Manchester	1
Puerto Rico	Rincón	2		Winchester	1
Mexico	Zihuatanejo	1		Swansea	1
Canada	Halifax	1		Bristol	1
	Vancouver	1		Glasgow	1
Indonesia	Padang	1		Edinburgh	1
	Merapi Volcano	2	Thailand	Pakarang Cape	1
	Pacitan	1	Peru	Callao/La Punta	1
	Banda Aceh	1	Vietnam	Nha Trang	1
USA	Oregon	5		Danang	4
	California	1	Pakistan	Unspecified	1
Spain	Marbella	1			

In the context of geographical regions, it is also worth noting that geographical regions may not be limited to the coastal part of the region. It seems that a part of the seabed is often included as well because it may provide important, more detailed information about tsunami wave propagation and parameters. Nonetheless, these more specific details are covered by item 7 in List B, which is focused on various data sources or datasets used for the initial configuration of the model parameters. Therefore, this aspect was not included in this part of the analysis. In almost every ABM included in the full-text analysis (see Sect. 2), a more specific part of the selected national state was used, that is, the specification of the geographic area usually has the structure of a nation-national region. Because this seems to be the most frequently used academic approach, this study adopts the same method.

### Item B2: What is the general purpose of the model?

The model attributes describe the major aim or purpose of the model. This review is focused on evacuation models and is therefore the joint purpose of all models in this review, despite significant variations in how the topic is handled. These differences are indicated in the model attributes.

The results of the analysis indicate that many models are focused on one of three approaches, which can be distinguished by their temporal aspects (which phase of evacuation the model is primarily focused on). These three approaches are (1) preparation and planning (e.g., urban planning, various tools to improve the recognition of tsunami-related risks), (2) the evacuation process itself (during the tsunami wave impact), and (3) optimization and improvement (application of risk/damage mitigation measures as a result of previous experience or knowledge). Other than these three major categories, there are other factors at play, although many more models can be added to one or more of these groups (Table 6).

**Table 6:** General model purpose or research areas

General purpose	Number
Behavior	11
Evac plans	10
Pedestrian evac	9
Urban planning/signs placement	8
Route optimization/pathfinding	6
Transportation network	6
Multiple/scalable/modular models	5
Mass evac	3
Decision support/guidance/decision making	3
Disaster management/planning	3
Multimodal transportation	3
Earthquake + tsunami	2
Focus on tourists/visitors	2
GIS-based ABM	2
Parallel comp	2
Risk evaluation and analysis	2
Traffic simulation	1
Case studies comparison	1
Evac combined with COVID-19	1
Building damage	1
Use of UAV	1

### Item B3: Are any special features/aspects/issues of evacuation explored?

Many models have special characteristics that can be of further interest. For example, along with the evacuation problem itself, the model can also work with issues related to traffic, multimodal transportation, COVID-19, debris, and building damage, among others. Therefore, this attribute helps to further distinguish individual models from each other and provides more information to the reader.

The most frequently researched aspect found is the study of individual behaviors. This is usually related to evacuation planning issues, and several studies have used scalable models that combine micro- and macro-scale perspectives. Another branch of research is focused on the environment, using approaches such as evacuation sign placement or urban planning and a design used to minimize the evacuation times. This is connected to transportation optimization and efficient path planning. GIS-based data are often used as a base layer for an environmental representation (Table 7).

**Table 7:** Specialized research topics of papers

Special feature	Number
Individual behaviors	8
Sign placement for evac	6
GIS-based data	5
Urban design	5
Emergency/evac planning	4
Scalable or modular models	4
Least-cost distance + ABM	3
Traffic-related	3
Transport networks	3
HPC or parallel computing	2
Impact of debris	2
Route optimization	2
Use of UAV	2
Vehicles in evac	2
Building damage	1
Case studies	1
Daily activities of agents	1
Game theory	1
Life Safety Model (LSM)	1
Radar vision	1
Sensitivity analysis	1
Tsunami + COVID-19	1
Use of safe areas	1

### Item B4: How is the environment represented?

There were significant differences in the representation of the environment. Some models use a simplified version of such a representation, and others use quite an elaborate environment model. This varies greatly among the models used; for example, bordered regions using only statistics, scaling up to highly detailed models with complex simulations of the hydrodynamics of tsunami wave advancement.

The most frequently used approach is to create a model using real map data, such as models based on GIS data, networks, or roads, which are derived from standard navigation application data (usually for land traffic, and quite less frequently for maritime traffic). More formal models use discrete mathematical representations, such as network graphs or grid-based approaches.

Another specific group consists of models focused on interior representations rather than maps or larger geographical areas. Although such an approach is usually related to highly detailed mechanisms for the simulation of building damage, interior evacuations, and other factors, surprisingly, several examples of combinations of interior/exterior perspectives were found as well. In these cases, exterior areas are usually limited to city districts, tourist venues, or other similarly limited exterior spaces (Table 8).

**Table 8:** Number of models using various types of environment representations

Environment representation	Number
GIS-data based	13
City or region map	12
Network (roads)	10
Unspecified	5
Network (graph)	4
Grid-based	3
Combined interior + exterior	3
Building plan	2
Table	1

### Item B5: What types of agents are defined, what are their attributes?

Because all models included in this study are to a smaller or larger extent related to evacuation issues, there is usually some representation of the evacuees included. However, many other entities may be represented in the model as agents. This attribute is quite important for the differentiation of the individual models because it is closely related to the complexity of the multi-agent systems used. Again, the complexity varies from a highly simplified representation and uncomplicated agent architectures to complex models incorporating social aspects of behavior and mutual interactions.

To date, the type of agent that is most frequently used is typically a form of pedestrian representation, which is combined with vehicles in certain studies. Quite often, various types of individuals are further distinguished to capture differences between local populations, tourists, evacuees, and other individuals (Table 9).

**Table 9:** Specific types of agents used in individual studies

Type of agent	Number
Pedestrians	21
Pedestrians + vehicles	9
Evacuees	9
Unspecified	4
Vehicles	4
General population	3
Individuals + families	2
Multiple models	1

### Item B6: What are the agents’ attributes, if specified?

The complexity of the model is often reflected in the number of individual attributes incorporated in the model. The purpose of this attribute is to reflect this complexity by providing a general description of the aspects of this model. In some cases, it was impossible to obtain a complete list of attributes, which were often not listed in the papers covered in this study. However, the number of individual attributes is an important aspect that reflects the complexity of the model, and as such, it was included as one of the monitored characteristics.

Although many papers do not list agent-related attributes specifically, the most frequently used factor is speed. Given the fact that there is generally a temporal limitation for evacuation procedures, this is quite logical. Speed is often combined with position, location, and direction. It can be assumed that the number of papers working with position attributes is actually higher, although this is usually not specifically mentioned in the literature. Other personal characteristics also seem to play important roles here, i.e., age, role, psychological aspects (often related to decision making), gender, and field of vision. Several studies used a large number of descriptive attributes in the models, going as far as listing over 50 attributes to provide an example (Table 10).

**Table 10:** List of agent-related attributes

Agent attributes	Number
Speed	22
Unspecified	14
Age	12
Position/location	9
Temporal factors	8
Agent type/status/role	7
Multiple factors	5
Direction	4
Psychological factors	4
Vision	3
Gender	3
Method of transport	3
Car/pedestrian movement attributes	3
Evac point/shelter attributes	3
Dependent population nearby	2
Decision making	2
Crowd density/distance to others	2
Size	1
Activities/actions	1

### Item B7: What input data/variables are used in the models?

Models are often constructed on reliable real-world datasets or input data. This is a crucial factor when evaluating the validity of the model because randomly generated data tend to provide less reliable results. Another factor that makes this attribute of the model important is the ability to reproduce the experiments.

As can be expected, there is a frequent use of GIS-related data or standard formats of regional navigational maps, known from normally used navigation devices. However, the most frequently used characteristics are individual physical attributes and a speed of movement description provided in the models. This correlates with the analysis of item B6 (see Sect. 4.6), in which speed plays the most important role as well. Many models use census data and surveys or official data provided by various official organizations related to tsunami impact mitigation efforts, which generally have a positive impact on the model validity (Table 11).

**Table 11:** Sources of input data or variables in ABMs

Input used	Number
Individual physical attributes, speed	23
GIS, regional maps	18
Routes	15
Individual behavior and decision making	12
Census data and surveys	11
Spatial distribution	10
Population distribution unspecified/random	8
Unspecified	7
OpenStreetMap	6
Probabilistic distribution	4
Buildings, buildings floor plans	4
Graph representation	4
HAZUS	3
SHOA	3
Vehicular movement data	3
GSI data	2
CDMC data	2
Evacuees	2
NSCRD	1
ONEMI	1
GeoClaw	1
INEGI	1
CRED database	1
Mobile devices data	1

### Item B8: What output data/variables/measures does the model produce?

The models vary in complexity, level of detail, number of agents, and other aspects, and therefore produce various types of output data. This is another important attribute of a model because it straightforwardly reflects its purpose and indicates the key performance factors being measured.

The results showed that the most frequently used output parameters were the number of people saved or lost and the evacuation time/speed. Because many models are focused on optimizing evacuation routes, these factors in the form of traffic network optimization or the use of shortest paths to safe zones are used as output factors as well (Table 12).

**Table 12:** Output data

Output type	Number
People saved	15
Time limits/evac time	15
People lost	13
Traffic networks balancing parameters	5
Shortest path/evac path	3
Evacuee status	3
HPC load balancing/scalability	2
Multiple factors	2
Flow-related factors	2
Infection exposition	1
Countermeasure effectiveness	1
Behavioral consequences	1
Building damage	1
Risk assessment/analysis	1
Spatial integration rate	1
Capacity demand index	1
Movement direction	1

### Item B9: Are there any simplifications or features omitted from the model and/or proposed by the authors for future work?

The models presented in this paper are commonly the results of ongoing long-term research and are becoming more complex and elaborate over time. This attribute serves as an indication of what is being either intentionally omitted from the model or intended for implementation in future research.

Although the majority of studies do not mention any simplifications specifically, when they are mentioned, there is a large variability in the topics. The most frequently mentioned is the incorporation of more detailed social mechanics into the models. Other factors and future topics of research are too varied to show some common trends (Table 13).

**Table 13:** Simplifications or topics for future study

Type of simplification/omitted factors	Number
N/A of unspecified	27
Social aspects or behavior	7
Shortest path	3
Population distribution	3
Transportation details	3
Vehicles excluded	2
Uniform probabilities	2
Hydrodynamics model	2
Decision-making-related aspects	2
Precise official information	1
Multiple factors combination	1
Specific evacuation routes	1
Individual perception	1
Physical interactions	1
Debris	1
Large-scale areas	1
Geographical details	1
Real-time data	1

### Item B10: Are there any specific algorithms used by agents?

In many cases, agents implemented in the model use standard, well-known algorithms for some of their behavioral components, such as pathfinding, coordination, movement, and communication protocols. Because this can be an important factor for differentiation between models, it is one of the monitored model attributes.

In the majority of studies, no specific algorithms were mentioned. When they are used, they are usually related to pathfinding and path planning approaches. This correlates with the attributes mentioned above, where speed and evacuation times seem to be the most crucial performance indicators in the models (Table 14)..

**Table 14:** Specific algorithms used in ABMs

Algorithm/approach	Number
N/A or unspecified	25
Dijkstra	9
Rayleigh distribution	5
Linear programming evaluation	3
A*	3
Monte-Carlo	3
ORCA	3
Random walk	2
Shortest path	2
Markov chains	1
Naismith’s rule	1
Stable marriage problem	1
Genetic algorithms	1
Depth–velocity product	1

## Conclusions

Our paper critically assessed the latest papers on agent-based models of human response to natural disasters, namely tsunamis. Typically, the ABM approach was adopted to represent the evacuation process, exploration of crowd behavior during a natural disaster (earthquake and subsequent tsunami), evacuation planning and optimization, and estimation of casualties. Typical input variables are the magnitude and spatial distribution of shelters, distances or zones, size of the population, and categories (e.g., locals or tourists and children or adults).

The output variables are the evacuation time, number of casualties and survivors, and optimal evacuation routes (in the case of a comparison of the scenarios).

Surprisingly, the papers did not follow the agent-based modeling approach and standards. From a methodological perspective, applied models are not as systemic as one would expect based on experience from other domains (Bureš 2006). Moreover, the models are not described in a particularly sophisticated way: the ODD protocol was not broadly adopted, and validation and verification methods were not systematically applied. Documentation and source codes were also not provided by the authors of the models; therefore, replication of the experiments is nearly impossible.

The models typically illustrate the phenomena using a selected algorithm; for example, pathfinding to shelters was frequently examined using Dijkstra’s method. Few models focus on specific issues, such as exploration of COVID transmission during a tsunami evacuation in the lockdown of the city (Callejas et al. 2020) or exploration of the performance and scalability of an agent-based mass tsunami evacuation simulation within high-performance and distributed computing (Aguilar et al. 2017).

In general, these models illustrate the significant potential of an agent-based approach in relation to the exploration of natural hazards; however, their achievements are insufficient.

Through a systematic analysis of relevant sources from the subject domain, we have identified the following two directions in research, which have not yet been presented and will become the motivation for our further study:

1. Specifications of the large-scale agent-based metamodel of the tsunami are necessary. The metamodel integrates precise environmental/hydrodynamical flooding models with models of human response (immediate response such as crowd evacuation as well as long-term plans and measures to mitigate potential hazards).
2. Development of agent-based simulations in massive multi-user online map-based game frameworks supporting 3D graphics. The inspiration here comes from (Massey et al. 2018) and (Cheliotis 2021).

## Supplementary Information

Below is the link to the electronic supplementary material.

Supplementary file1 (PDF 209 kb)

## Data Availability

No code or software has been developed for this research.
