# Supplementary material for: Agent-based models of human response to natural hazards: systematic review of tsunami evacuation
Source: Nat Hazards (Dordr). 2022 Oct 5;115(3):1887–908. doi: 10.1007/s11069-022-05643-x (PMC9533266; doi:10.1007/s11069-022-05643-x)
Supplement: Supplementary file 1 — Supplementary file1 (PDF 209 kb) [file 11069_2022_5643_MOESM1_ESM.pdf]

Article title: Agent-Based Models of Human Response to Natural Hazards: Systematic Review of Tsunami Evacuation
Journal name: Natural Hazards
Authors:
Karel Mls – corresponding author, Faculty of Informatics and Management, University of Hradec Králové, 500 03 Hradec Králové, Czech Republic, ORCID(s): 0000-0002-7681-8277, e-mail: karel.mls@uhk.cz
Milan Kořínek, Kamila Štekerová, Petr Tučník, Vladimír Bureš, Pavel Čech, Martina Husáková, Peter Mikulecký, Tomáš Nacházel, Daniela Ponce, Marek Zanker, František Babič and Ioanna Triantafyllou

Online Resource 1. List of selected papers and their quantitative characteristics

No.	Reference	Sensitivity analysis is specified	Model validation is specified	Specification of validation method	Experimental scenarios are defined	Experiments are presented	Statistical methods are applied	ODD and source code are presented	Software is specified	Geographical area
1	Aguilar, L., Lalith, M., Ichimura, T., & Hori, M., 2017. On the performance and scalability of an HPC enhanced Multi Agent System based evacuation simulator. Procedia Computer Science, 108, 937-947.	no	no	no	no	only final results	no	source code	unspecified	large urban domains (hundreds km2), Kochi City, Japan

2	Alam, M. D. J., & Habib, M. A., 2020. Modeling Traffic Disruptions during Mass Evacuation. Procedia Computer Science, 170, 506-513.	yes	reference to another paper	reference to another paper	yes	final results only	no	no	Rstudio	Halifax Regional Municipality, Canada
3	Callejas, E., Inostrosa-Psijas, A., Moreno, F., Oyarzun, M., & Carvajal-Schiaffino, R., 2020. COVID-19 Transmission during a Tsunami Evacuation in a Lockdown City. 39th International Conference of the Chilean Computer Science Society (SCCC), 2020, pp. 1-8.	no	no	no	yes	yes	no	no	Gama Simulator	Iquique, northern Chile
4	Castro, S., Poulos, A., Herrera, J. C., & de la Llera, J. C., 2019. Modeling the impact of earthquake-induced debris on tsunami	no	no	comparison with real world data	yes	yes	yes	no	unspecified	Iquique, Chile

	evacuation times of coastal cities. Earthquake Spectra, 55(1), 137-158.									
5	Castro, S., Poulos, A., Urrutia, A., Herrera, J. C., Cienfuegos, R., & De La Llera, J. C., 2018. Impact of earthquake magnitude on the estimation of tsunami evacuation casualties. Proceedings of the 11th National Conference in Earthquake Engineering	no	no	no	yes	yes	yes	no	unspecified	Iquique, Chile
6	Faucher, J. E., Dávila, S., & Hernández-Cruz, X., 2019. A hybrid pedestrian evacuation model for tsunamis. IIE Annual Conference. Proceedings (pp. 982-987). Institute of Industrial and Systems Engineers (IISE).	no	no	no	yes	yes	yes	no	QGIS (r.walk]	Rincón, Puerto Rico

7	Faucher, J. E., Dávila, S., & Hernández-Cruz, X., 2020. Modeling pedestrian evacuation for near-field tsunamis fusing ALCD and agent-based approaches: A case study of Rincón, PR. International Journal of Disaster Risk Reduction, 49.	no	yes	comparison with other models	Y	yes	yes	no	QGIS (r.walk]	Rincón, Puerto Rico
8	Hatayama, M., Kosaka, T., & Hernandeze, A. H., 2019. Analysis on Tsunami Evacuation Options with Agent-based Simulation in Tourist Area. 2018 5th International Conference on Information and Communication Technologies for Disaster Management (ICT-DM), 2018, pp. 1-8.	no	no	no	yes	yes	no	no	DiMSIS	Zihuatanejo, Mexico

9	Helton, W. S., Kemp, S., & Walton, D., 2013. Individual differences in movements in response to natural disasters: Tsunami and earthquake case studies. Proceedings of the Human Factors and Ergonomics Society Annual Meeting, 57(1).	no	no	no	no	no	no	no	no	Christchurch
10	Ishida, R., Izumi, T., & Nakatani, Y., 2013. Simulation system of tsunami evacuation behavior during an earthquake around JR Osaka station area. CENTRIC 2012 : The Fifth International Conference on Advances in Human-oriented and Personalized Mechanisms, Technologies, and Services, 67-78.	no	no	no	yes	yes	no	no	no	Osaka city - Japan

11	Ishida, Y., & Hashimoto, S., 2015. Asymmetric Characterization of Diversity in Symmetric Stable Marriage Problems: An Example of Agent Evacuation. Procedia Computer Science, 60, 1472-1481.	no	no	no	yes	yes	no	no	no	X
12	Ito, E., Kawase, H., Matsushima, S., & Hatayama, M., 2020. Tsunami evacuation simulation considering road blockage by collapsed buildings evaluated from predicted strong ground motion. Natural Hazards, 101(3), 959-980.	no	building models Y, rest only briefly through references	no	yes	yes	no	no	Aritsoc and DiMSIS	Tanabe City in Wakayama Prefecture in Japan,Kamiyashiki and Katamachi
13	Jumadi, Carver, S., & Quincey, D. 2016. A Conceptual Framework of	no	no	no	no	yes	no	no	AnyLogic	Merapi volcano, Indonesia

	Volcanic Evacuation Simulation of Merapi Using Agent-based Model and GIS. Procedia - Social and Behavioral Sciences, 227, 402-409.									
14	Karbovskii, V., Voloshin, D., Karsakov, A., Bezgodov, A., & Zagarskikh, A., 2015. Multiscale Agent-based Simulation in Large City Areas: Emergency Evacuation use Case. Procedia Computer Science, 51, 2367-2376.	no	no	no	no	yes	no	no	PULSE	Vasilyevsky Island, St. Petersburg, Russia
15	Karbovskii, V. A., Voloshin, D. V., Puzyreva, K. A., & Zagarskikh, A. S., 2014. Personal Decision Support Mobile Service for Extreme Situations.	no	no	yes	comparsion with real world data	no	yes	no	unspecified	X

	Procedia Computer Science, 29, 1646-1655.									
16	Katayama, K., Takahashi, H., Yokota, N., Sugiyasu, K., Kitagata, G., & Kinoshita, T., 2019. An Effective Multi-UAVs-Based Evacuation Guidance Support for Disaster Risk Reduction. *2019 IEEE International Conference on Big Data and Smart Computing (BigComp)*, 2019, pp. 1-6.	no	yes	Comparison with real world data	no	yes	no	no	Dash	X
17	Kunwar, B., Simini, F., & Johansson, A., 2014. Large Scale Pedestrian Evacuation Modeling Framework Using Volunteered Geographical Information. Transportation Research Procedia, 2, 813-818.	yes	no	no	yes	yes	yes	no	unspecified	multiple locations (Bath and North East Somerset, Chichester, Wakefield, Stirling Council, Milton Keynes, Bradford,

										Manchester, Winchester, Swansea, City of Bristol, Glasgow City, City of Edinburgh)
18	Le, V. M., Chevaleyre, Y., Vinh, H. T., & Zucker, J. D., 2015. Hybrid of linear programming and genetic algorithm for optimizing agent-based simulation. Application to optimization of sign placement for tsunami evacuation. *The 2015 IEEE RIVF* International Conference on Computing & Communication Technologies - Research, Innovation, and Vision for	yes	no	no	no	yes	no	no	GAMA	Danang - Vietnam

	Future (RIVF), 2015, pp. 138-143									
19	Le, V. M., Chevaleyre, Y., Zucker, J. D., & Vinh, H. T,. 2013. Speeding up the evaluation of casualties in multi-agent simulations with Linear Programming application to optimization of sign placement for tsunami evacuation. The 2013 RIVF International Conference on Computing & Communication Technologies - Research, Innovation, and Vision for Future (RIVF), 2013, pp. 215-220	yes	yes	no	yes	yes	no	no	GAMA	Danang - Vietnam
20	Le, V. M., Chevaleyre, Y., Zucker, J. D., Vinh, H. T., 2014. Approaches to	yes	no	no	yes	yes	no	no	GAMA	Danang - Vietnam

	optimize local evacuation maps for helping evacuation in case of Tsunami. 196, 21-31.									
21	Le, V. M., Vinh, H. T., Zucker, J. D., 2017. Reinforcement learning approach for adapting complex agent-based model of evacuation to fast linear model. Seventh International Conference on Information Science and Technology (ICIST), 2017, pp. 369-375	yes	no	no	yes	yes	yes	no	GAMA	Danang - Vietnam
22	León, J., Castro, S., Mokrani, C., & Gubler, A., 2020. Tsunami evacuation analysis in the urban built environment: a multi-scale perspective through two modeling approaches in	no	yes	no	yes	yes	yes	no	GIS, unspecified	Great Valparaiso Metropolitan Area + Viña del Mar, Chile

	Viña del Mar, Chile. Coastal Engineering Journal, 62(3), 389-404.									
23	León, J. and March, A., 2014. An urban design framework for tsunami evacuation safety: A case study of two Chilean cities. León, J., and A. P. March. Proceedings of the 5th International Disaster and Risk Conference: Integrative Risk Management—The Role of Science, Technology and Practice, Davos, Switzerland, pp. 24-28. 2014.	no	no	no	yes	no	no	no	Agent Analyst, Repast, ArcGIS	Iquique +Talcahuano, Chile
24	León, J., March, A., 2014. Urban morphology as a tool for supporting tsunami rapid resilience.A case study of	no	no	no	yes	yes	yes	no	Agent Analyst, Repast, ArcGIS	Talcahuano, Chile

	Talcahuano, Chile. Habitat International, 43, 250-262.									
25	León, J., & March, A., 2016. An urban form response to disaster vulnerability: Improving tsunami evacuation in Iquique, Chile. Environment and Planning B: Planning and Design, 43(5), 826-847.	no	no	no	yes	yes	yse	no	Agent Analyst, Repast, ArcGIS	Iquique , Chile
26	León, J., Mas, E., Catalán, P. A., Moya, L., Gubler, A., Koshimura, S., Cienfuegos, R., 2021. Development of calibrated tsunami evacuation models through real-world collected data: The case study of Coquimbo-La Serena, Chile. *IOP Conf. Ser.: Earth Environ. Sci.* 630 012005.	N	N	comparison of ABMS with mobile phone company data to calibrate the outomes of ABM	Y	no	yes	no	PARI-AGENT, STOC-ML	Coquimbo-La Serena, Chile

27	León, J., Mokrani, C., Catalán, P., Cienfuegos, R., & Femenías, C., 2018. Examining the role of urban form in supporting rapid and safe tsunami evacuations: a multi-scalar analysis in Viña del Mar, Chile. Procedia Engineering, 212, 629-636.	no	no	no	yes	yes	no	no	PARI-AGENT	city of Vina del Mar, Chile
28	Makinoshima, F., Imamura, F., Abe, Y., 2018. Enhancing a tsunami evacuation simulation for a multi-scenario analysis using parallel computing. Simulation Modelling Practice and Theory, 83, 36-50.	future work	yes	yes	vague description	no	no	no	no	Ksennuma City, Japan
29	Mas, E., Koshimura, S., Imamura, F., Suppasri, A., Muhari, A., Adriano, B., 2015. Recent Advances in	no	no	no	vague description	described in general	no	no	MATSim	Arahama, Japan, Padang, Indonesia, Pakarang Cape, Thailand, Natori,

	Agent-Based Tsunami Evacuation Simulations: Case Studies in Indonesia, Thailand, Japan and Peru. Pure and Applied Geophysics, 172(12), 3409-3424.									Japan, Case of La Punta, Peru
30	Medina, N., Sanchez, A., Vojinovic, Z., 2016. The Potential of Agent Based Models for Testing City Evacuation Strategies Under a Flood Event. Procedia Engineering, 154, 765-772.	no	no	no	no	no	no	no	Repast Symphony, Java	Marbella, Spain
31	Mostafizi, A., Wang, H., Cox, D., Cramer, L. A., Dong, S., 2017. Agent-based tsunami evacuation modeling of unplanned network disruptions for evidence-driven resource allocation and retrofitting	yes	no	no	yes	yes	yes	no	NetLogo, GIS, RNetLogo, R	Seaside, Oregon

	strategies. Natural Hazards, 88(3), 1347-1372.									
32	Mostafizi, A., Wang, H., Cox, D., Dong, S., 2019. An agent-based vertical evacuation model for a near-field tsunami: Choice behavior, logical shelter locations, and life safety. International Journal of Disaster Risk Reduction, 34, 467-479.	no	no	no	one scenario only	yes	no	no	NetLogo, R	dtto
33	Mostafizi, A., Wang, H., Dong, S., 2019. Understanding the Multimodal Evacuation Behavior for a Near-Field Tsunami. Transportation Research Record. 2019;2673(11):480-492.	yes	no	no	varying parameters	yes	no	no	NetLogo	dtto

34	Nakanishi, H., Wise, S., Suenaga, Y., Manley, E., 2020. Simulating emergencies with transport outcomes Sim (SETOSim): Application of an agent-based decision support tool to community evacuation planning. International Journal of Disaster Risk Reduction, 49.	no	no	no	no	yes	no	ODD, source code	MASON, Java	Takamatsu City, Japan
35	Naqvi, A., 2017. Deep Impact: Geo-Simulations as a Policy Toolkit for Natural Disasters. World Development, 99, 395-418.	yes	no	no	yes	yes	no	source code	NetLogo	northern Pakistan
36	Nguyen, T. N. A., Zucker, J. D., Nguyen, M. H., Drogoul, A., Nguyen, H. P., 2012. Simulation of emergency evacuation of pedestrians along the road	no	no	no	no	yes	no	ODD	no	Nhatrang, Vietnam

	networks in Nhatrang city. IEEE RIVF International Conference on Computing & Communication Technologies, Research, Innovation, and Vision for the Future, 2012, pp. 1-6.									
37	Poulos, A., Tocornal, F., de la Llera, J. C., Mitrani-Reiser, J., 2018. Validation of an agent-based building evacuation model with a school drill. Transportation Research Part C: Emerging Technologies, 97, 82-95.	Y	video analysis	video analysis	no	yes	no	algorithm in pseudocode	NetLogo	Iquique, northern Chile
38	Sahal, A., Leone, F., Péroche, M., 2013. Complementary methods to plan pedestrian evacuation of the French Riviera's beaches in case of tsunami threat: Graph-and multi-	no	yes	comparison to real workd data	yes	yes	no	no	SimWalk	French Mediterranean coast

	agent-based modelling. Natural Hazards and Earth System Sciences, 13(7), 1735-1743.									
39	Slucki, A., Nielek, R., 2015. Advantages of cooperative behavior during Tsunami evacuation, 9021, 203-212.	no	no	no	yes	yes	no	no	NetLogo	Kamaishi, Japan
40	Solís, I. A., Gazmuri, P., 2017. Evaluation of the risk and the evacuation policy in the case of a tsunami in the city of Iquique, Chile. Natural Hazards, 88(1), 503-532.	yes	yes	yes	yes	yes	no	no	NetLogo, GIS	Iquique, Chile
41	Tagg, A., Davison, M., Wetton, M., 2016. Use of agent-based modelling in emergency management under a range of flood hazards. E3S Web Conference, 7 19006.	no	no	no	no	yes	no	no		East Coast, UK - Lincolnshire and Norfolk, Humberside

42	Takabatake, T., Esteban, M., Nistor, I., Shibayama, T., Nishizaki, S., 2020. Effectiveness of hard and soft tsunami countermeasures on loss of life under different population scenarios. International Journal of Disaster Risk Reduction, 45.	no	no	no	yes	yes	yes	no	Artisoc 4.0	Yuigahama Beach, Kamakura, Japan
43	Takabatake, T., Fujisawa, K., Esteban, M., Shibayama, T., 2020. Simulated effectiveness of a car evacuation from a tsunami. International Journal of Disaster Risk Reduction, 47.	no	yes	comparison with real data (traffic jams observed, Tohoku Earthquake Tsunami)	yes	yes	yes	no	Artisoc	Shinguu City, Japan
44	Takabatake, T., Nistor, I., St-Germain, P., 2020. Tsunami evacuation simulation for the District of Tofino, Vancouver Island,	yes	yes	comparison with inundation data from Tohoku	yes	yes	yes	no	Artisoc	Tofino, Vancouver, Canada

	Canada. International Journal of Disaster Risk Reduction, 48.									
45	Takabatake, T., Shibayama, T., Esteban, M., Ishii, H., 2018. Advanced casualty estimation based on tsunami evacuation intended behavior: case study at Yuigahama Beach, Kamakura, Japan. Natural Hazards, 92(3), 1763-1788.	no	future work	no	yes	yes	yes	no	Artisoc 4.0	Kamakura, Japan
46	Takabatake, T., Shibayama, T., Esteban, M., Ishii, H., Hamano, G., 2017. Simulated tsunami evacuation behavior of local residents and visitors in Kamakura, Japan. International Journal of Disaster Risk Reduction, 23, 1-14.	no	no	no	yes	yes	yes	no	Artisoc 4.0	Kamakura, Japan

47	Usman, F., Murakami, K., Dwi Wicaksono, A., Setiawan, E., 2017. Application of Agent-Based Model Simulation for Tsunami Evacuation in Pacitan, Indonesia. MATEC Web of Conferences 97, 01064.	no	no	comparison of ABMS and Shelter Plan Analysis	yes	no	yes	yes	Repast Simphony, ARCGis	Pacitan City, Indonesia
48	Wafda, F., Saputra, R. W., Nurdin, Y., Nasaruddin, Munadi, K., 2013. Agent-based tsunami evacuation simulation for disaster education. International Conference on ICT for Smart Society, 2013, pp. 1-4.	no	no	no	yes	yes	no	ODD	NetLogo	Banda Aceh, Indonesia
49	Wang, H., Mostafizi, A., Cramer, L. A., Cox, D., Park, H., 2016. An agent-based model of a	yes	no	no	yes	yes	no	no	NetLogo	Seaside, Oregon

	multimodal near-field tsunami evacuation: Decision-making and life safety. Transportation Research Part C: Emerging Technologies, 64, 86-100,.									
50	Wang, Z., Jia, G., 2021. A novel agent-based model for tsunami evacuation simulation and risk assessment. Natural Hazards, 105(2), 2045-2071.	no	no	no	yes	yes	no	no	NetLogo, R	Seaside, Oregon
51	Wijerathne, L., Petprakob, W., Aguilar, L., Hori, M., Ichmura, T., 2018. Scalable HPC Enhanced Agent Based System for Simulating Mixed Mode Evacuation of Large Urban Areas. Transportation Research Procedia. 34. 275-282.	no	no	no	yes	yes	no	no	unspecified	Central Tokyo, Japan

52	Wijerathne, M. L. L., Melgar, L. A., Hori, M., Ichimura, T., Tanaka, S., 2013. HPC Enhanced Large Urban Area Evacuation Simulations with Vision based Autonomously Navigating Multi Agents. Procedia Computer Science, 18, 1515-1524.	no	yes	no	yes	yes	no	source code	C++	Kochi city
53	Wood, N., Henry, K., Peters, J, 2020. Influence of demand and capacity in transportation simulations of short-notice, distant-tsunami evacuations. Transportation Research Interdisciplinary Perspectives, 7.	no	no	no	yes	yes	yes	no	MATSim	California, Aleutian
